# Mesenteric Involvement in Large‐Vessel Giant Cell Arteritis: Why PET‐CT Findings Should Be Paired With Objective Ischemia‐Risk and Gastrointestinal Follow‐Up Reporting

**DOI:** 10.1002/ccr3.73180

**Published:** 2026-07-16

**Authors:** Muhammad Abdullah Awan, Hammad‐ud‐din Raza

**Affiliations:** ^1^ Wah Medical College National University of Medical Sciences Pakistan

**Keywords:** functional gut syndrome, giant cell arteritis, large‐vessel vasculitis, mesenteric ischemia, PET‐CT, tocilizumab

## Abstract

PET‐CT is valuable for identifying large‐vessel giant cell arteritis with mesenteric involvement, but case‐based learning is strengthened when vascular uptake is paired with objective ischemia‐risk assessment, gastrointestinal monitoring, and longer follow‐up. Future reports should distinguish mesenteric arteritis, bowel ischemia, colitis, and functional gastrointestinal symptoms with reproducible clinical, biochemical, endoscopic, and imaging endpoints.

Dear Editor,

We read with great interest the case report by Rao and O'Connor [1], titled “Giant Cell Arteritis With Mesenteric Involvement and Superimposed Functional Gut Syndrome: A Case Report,” published in Clinical Case Reports. The authors describe a 69‐year‐old woman with a 6‐month history of progressive postprandial abdominal pain, weight loss, markedly elevated inflammatory markers, and FDG PET‐CT evidence of large‐vessel giant cell arteritis (LV‐GCA) involving the thoracic and abdominal aorta, subclavian and axillary arteries, common carotids, common iliac arteries, and superior mesenteric artery (SMA) [1]. The report is valuable because mesenteric involvement in GCA is rare, diagnostically difficult, and potentially fatal if ischemia progresses to bowel infarction or perforation.

However, one point deserves stronger emphasis: PET‐CT evidence of vascular inflammation should be reported alongside objective markers of bowel ischemia risk. In the presented case, PET‐CT showed SMA involvement and diffuse colonic avidity, but contrast‐enhanced CT described patent splanchnic vessels; flexible sigmoidoscopy and colonoscopy did not demonstrate inflammatory changes, biopsies were negative, and later colonoscopy was limited by poor bowel preparation [1]. These findings make the case clinically instructive, but they also highlight the need to distinguish four overlapping possibilities: mesenteric arteritis, true bowel ischemia, inflammatory colitis, and persistence of functional gastrointestinal symptoms.

This distinction is clinically important because the management implications are not identical. Mesenteric arteritis on PET‐CT may identify high‐risk systemic vasculitis before infarction occurs, whereas established bowel ischemia requires closer surgical awareness, serial abdominal examination, lactate or acid–base assessment, vascular imaging, and low threshold for urgent escalation. In contrast, functional gut syndrome may coexist with LV‐GCA but should not be used to explain new red flags such as postprandial pain, food avoidance, constitutional symptoms, anemia, and extreme CRP/ESR elevation. Rao and O'Connor [1] appropriately emphasize that the patient's pre‐existing functional gut disorder contributed to diagnostic delay. Future reports could strengthen this lesson by explicitly identifying the “transition point” from a stable functional baseline to a systemic inflammatory vascular syndrome.

For future case reports of mesenteric LV‐GCA, we suggest a minimum ischemia‐risk dataset. This could include baseline and serial abdominal pain pattern, postprandial timing, food fear, weight trajectory, bowel habit change, lactate level where clinically available, acid–base status, abdominal examination findings, CT angiography or contrast CT features, vessel patency or stenosis, bowel wall thickening, pneumatosis, mesenteric edema, endoscopic findings, biopsy interpretation, and whether surgical or vascular consultation was considered. Such reporting would clarify whether the case represents inflammatory arterial involvement without ischemia, evolving chronic mesenteric ischemia, or symptomatic overlap with functional disease.

A second issue is diagnostic hierarchy when temporal artery biopsy is negative. The bilateral temporal artery biopsies in this case were performed on Day 7 after prednisolone commencement and showed no inflammation or giant cells [1]. This does not invalidate the diagnosis, especially because extracranial LV‐GCA may lack classic cranial findings and may be better captured by systemic imaging. Nevertheless, future reports would be clearer if they explicitly state how PET‐CT, inflammatory markers, clinical features, and response to immunosuppression were weighted when biopsy was negative. This is particularly important for readers working in settings where PET‐CT access, temporal artery ultrasound, or biopsy timing may differ.

A third point concerns tocilizumab safety and monitoring. The authors note that tocilizumab was commenced to facilitate steroid weaning after limited symptomatic improvement with corticosteroids, and they appropriately mention the small but significant risk of bowel perforation with IL‐6 inhibition, particularly with high‐dose prednisolone [1]. In a patient with mesenteric involvement and colonic PET avidity, future reports should describe the abdominal monitoring plan before and after IL‐6 blockade: warning symptoms explained to the patient, escalation thresholds, bowel‐perforation risk review, assessment for diverticular disease or occult colitis when possible, and how disease activity was followed once CRP became less reliable under IL‐6 inhibition.

Finally, follow‐up duration matters in this phenotype. Rao and O'Connor [1] report stability at 2 months, which is reassuring. Yet mesenteric LV‐GCA is a high‐risk vascular presentation, and short‐term symptomatic improvement does not fully define relapse risk, vascular remodeling, or late intestinal complications. Longer follow‐up with symptom scores, inflammatory markers interpreted in the context of tocilizumab, steroid dose, repeat vascular imaging when indicated, nutritional recovery, and absence of ischemic events would make the clinical outcome more reproducible.

Importantly, our comments do not weaken the authors' conclusion that PET‐CT is valuable in diagnostically challenging extracranial GCA. Rather, they extend the central clinical message of the case: when functional gastrointestinal disease and mesenteric vasculitis overlap, clinicians need structured red‐flag recognition and objective follow‐up endpoints. This would help future readers distinguish radiologic mesenteric involvement from clinically significant bowel ischemia and avoid both diagnostic under‐recognition and over‐attribution of chronic symptoms to vasculitis.

This case usefully highlights a rare presentation of LV‐GCA with mesenteric involvement and superimposed functional gut syndrome. Future reports should pair PET‐CT vascular findings with objective ischemia‐risk assessment, clear diagnostic hierarchy when temporal artery biopsy is negative, tocilizumab‐specific gastrointestinal safety monitoring, and longer longitudinal follow‐up. These details would transform a rare case observation into a more reproducible clinical framework for managing mesenteric involvement in GCA.

Sincerely,

Muhammad Abdullah Awan

## Author Contributions

**Muhammad Abdullah Awan:** conceptualization, data curation, formal analysis, project administration, writing – original draft, writing – review and editing. **Hammad‐ud‐din Raza:** data curation, formal analysis, writing – original draft, writing – review and editing.

## Funding

The authors have nothing to report.

## Ethics Statement

Ethics approval was not required for this Letter to the Editor because it discusses a previously published case report and does not include new patient data.

## Consent

Patient consent was not required for this Letter to the Editor because no new identifiable patient information is presented.

## Conflicts of Interest

The authors declare no conflicts of interest.

## Data Availability

Data sharing not applicable to this article as no datasets were generated or analysed during the current study.
